# We’re here too: child health information-seeking experiences and preferences of Red River Métis families – a qualitative study

**DOI:** 10.1186/s12939-023-02069-0

**Published:** 2023-12-06

**Authors:** Lisa Knisley, S. Michelle Driedger, Lisa Hartling, Frances Chartrand, Julianne Sanguins, Shannon D. Scott

**Affiliations:** 1 Children’s Hospital Research Institute of Manitoba, Winnipeg, Canada; 2 Faculty of Nursing, Edmonton Clinic Health Academy, University of Alberta, Level 3, 11405 87 Avenue, Edmonton, Canada; 3 Rady Faculty of Health Sciences, Department of Community Health Sciences, University of Manitoba, Winnipeg, Canada; 4 Alberta Research Centre for Health Evidence, Department of Pediatrics, University of Alberta, Edmonton, AB Canada; 5 Manitoba Métis Federation, Winnipeg, Canada

**Keywords:** Pediatrics, Information, Knowledge translation, Knowledge mobilization, Child health, Indigenous, Métis, Red River Métis

## Abstract

**Background:**

Red River Métis families need access to meaningful and appropriate resources when their children are sick. At the invitation of the Manitoba Métis Federation (MMF) to partner in this research, our aim was to understand Red River Métis parents’ experiences and preferences for seeking child health information when their child is acutely ill, to inform the adaptation of existing parent resources.

**Methods:**

A qualitative descriptive approach underpinned by a participatory paradigm guided this study. Semi-structured interviews were conducted with 19 Red River Métis parents and Elders via Zoom or telephone. An inductive thematic analysis approach was used to explore patterns and themes across the data.

**Results:**

Analysis generated four themes: (1) We’re here too; (2) We are not all the same; (3) Finding trustworthy information; and (4) Information needs to be widely available. Red River Métis pride was prominent in the results. Parents provided tangible ways to modify existing parent resources, including adding information on how to access Elders, healers and/or traditional medicines and showing different family structures, transport, living situations, Métis names, and incorporating Métis languages. While most parents reported looking for child health information online, they also stressed the need to provide multiple options, including information sheets, recognizing that parents seek information in different contexts. Parents also emphasized the importance of accessible, safe spaces to find child health information, including local schools, community centres, healthcare organizations and the MMF.

**Conclusion:**

There is a lack of child health information created specifically for Red River Métis families. The development of this information can support their information needs and preferences and the ongoing efforts to revitalize Red River Métis culture and language. Study findings will inform the adaptation and dissemination of existing child health resources to ensure they reflect Red River Métis parents’ information needs and preferences. This research is a critical step in addressing an identified need for Red River Métis families to have culturally relevant and meaningful child health resources, and in the pursuit of equitable care for all children in Canada.

**Trial Registration:**

N/A.

## Background

Worried families often look for health information to help decide how to care for their sick child [1–5], and what they find can have a crucial impact on a child’s health status [2]. Understanding and prioritizing families’ child health information needs can support their decision-making, healthcare access, and appropriate healthcare service delivery [6–10]. It is also a critical step in developing information that is accessible, useful and meaningful.

Knowledge translation (KT) “is the synthesis, dissemination, exchange, and ethically sound application of knowledge to improve health, provide more effective health services and products, and strengthen the health care system” [11]. Members of our research team have created successful KT tools (e.g., videos, infographics) with parents that merge evidence, personal narratives/stories and art to share information on common childhood conditions [12–17]. These resources were created to have broad accessibility and fit with the Canadian population. However, further research is needed on whether these resources are relevant for specific cultural groups and contexts [18–21].

This project arose from a need identified by the Manitoba Métis Federation (MMF; the national government of the Red River Métis) for Red River Métis families to have access to meaningful and appropriate resources when their children are sick. The MMF saw existing parent tools developed by Translating Emergency Knowledge for Kids (TREKK; trekk.ca), a knowledge mobilization network for children’s emergency care, and approached TREKK to fund the adaptation of these tools for Red River Métis families [22]. At the invitation of the MMF to partner in this research, our aim was to understand Red River Métis parents’ experiences and preferences for seeking child health information when their child is acutely ill. The research team consists of the MMF (FC, JS), a Red River Métis scholar (SMD) and allied academics from TREKK (LK, LH, SS).

The Canadian Constitution recognizes Métis, First Nations, and Inuit Peoples as the three Indigenous groups and first peoples of Canada [23]. As a people, the Métis were the initial off-spring of mixed First Nations and European ancestry in the 17th and 18th centuries with historical ties to the Red River Valley and westward expansion during the fur trade [24]. The Métis developed a united and unique nation in North America’s northwest, forming its own language, traditions, and culture [23, 25, 26]. Manitoba is widely recognized as the birthplace of the Red River Métis Nation [25, 27]. Although some Métis might adopt more Indigenous ways of knowing while others may follow more Western ways of knowing, Red River Métis remain in an interstitial space – as being neither First Nations nor European settler populations [28]. Like other Indigenous Peoples, Métis have experienced and continue to experience the legacy of colonial policies that attempted to remove them from Canada and disrupt their ways of knowing and being, [27] including sexual exploitation and violence against Métis women, residential schools, forced removal from homes and communities, the lawless administration of Métis lands, historical dispossession of lands and even outright war [29–31]. It took until 2013 for the Supreme Court of Canada to conclude the Canadian government failed its constitutional obligations towards the Métis following the creation of Manitoba, in 1870 [32].

The MMF represents Red River Métis in the Canadian province of Manitoba, as well as Citizens living outside of the province with historical connections to the Red River Settlement. In 2021, the number of self-identifying Métis living in Manitoba was 96,730 with 46.7% living in Winnipeg [33]. It is important to note that although individuals self-identify as métis, it does not mean they are recognized as Red River Métis citizens by the MMF. The MMF governance structure divides the province into seven regions, and within each region there are 135 smaller divisions referred to as Locals. Approximately half of the Red River Métis population live within the city of Winnipeg, Manitoba’s capital city, with the rest living diffusely throughout the rest of the province.

Red River Métis have expressed a need for reliable health information and that health systems have left much of their information needs unmet [29]. Métis parents have recognized culture and a positive sense of Métis identity as essential elements of raising children and as key components that are missing from health services [34]. Yet, Métis are underrepresented within research, [35, 36] and there is a silence in the literature about Métis health and well-being [27]. Furthermore, Red River Métis are rarely included in a distinctions-based approach. Most research that includes Red River Métis tend to be pan-Indigenous, grouping them with First Nations and Inuit, [26] which can diminish their unique and diverse experiences.

Red River Métis and First Nations Peoples often live side-by-side within Manitoba, yet have unequal access to health services and supports due to different fiduciary responsibilities between the federal and provincial governments, which can increase health inequities and challenge the uptake of research, policies, programming, and services [37]. Red River Métis access health services through the province the same as other Manitoba residents; they do not have access to federally supported health services available to on-reserve First Nations or Inuit.

Findings from this study will inform the adaptation [38, 39] and dissemination of existing child health resources to ensure they reflect Red River Métis parents’ information needs and preferences. Furthermore, understanding the barriers that prevent parents from getting the information they need can help expose and challenge existing colonial processes perpetuating inequitable care.

## Methods

A qualitative descriptive approach, [40, 41] underpinned by a participatory paradigm, [42, 43] guided this study. A decolonizing research framework [44] guided the use of ethical, iterative, culturally-based, and process-oriented methods that were relational, respectful, relevant, reciprocal and responsible [45]. The Consolidated Criteria for Reporting Qualitative Research checklist was used [46]. Meetings took place with MMF leadership to explore the adaptation of existing parent resources. Tri-Council Policy Statement [47] of ethical guidelines, and Ownership, Control, Access and Stewardship (OCAS) principles of the MMF [48] were followed. Ethics approval was obtained from the MMF and University of Alberta Health Research Ethics Board [#Pro00111866].

### Recruitment

Parents were invited to participate in a Zoom or telephone interview to increase feasibility of data collection during the COVID-19 pandemic. A recruitment poster and standardized email messages were developed and shared using established communication channels through the MMF. Recruiting took place from July 2021 to April 2022 via word-of-mouth or emails from the MMF and snowball sampling methods [49]. Parents were purposefully selected to aim for a breadth of age, gender and geographic representation [50]. Interested parents were instructed to contact the lead author (LK) via email or phone who then provided them with a study information sheet and determined eligibility: (a) self-identification as a Red River Métis citizen in Manitoba; (b) a parent (or guardian) of a child of any age; (c) fluent in English (speaking, hearing) to allow for meaningful participation in the interview. Elders, who are people that hold respected position within Red River Métis communities to provide guidance, share wisdom and pass knowledge of cultural importance from one generation to the next, were invited to participate through the MMF [51]. Participating Elders were also parents and grandparents.

### Data collection

To maintain consistency, LK coordinated and conducted each interview using a semi-structured interview guide developed in consultation with the research team. Interview topics included child health information needs and preferences, necessary changes to existing resources, trusted sources for health information, preferred topics and formats, and experiences finding the information. Eligible parents were contacted to schedule an interview via phone or Zoom [52] based on their preference. Parents were given the option to provide written or verbal consent. Consent forms (and the digital recordings for verbal consents) were stored, along with the transcription of the audio file, on a password protected secure, Canadian server.

Parents were asked to look at one or two resources available on trekk.ca in advance of the interview. Two parents did not review the web page due to lack of internet access. In these instances, the resources were described to the participant by LK. All interviews were digitally recorded through Zoom and professionally transcribed. Parents were asked to complete a demographic questionnaire (Table 1) at the end of the interview and given a gift card in recognition of the time, following MMF guidelines ($50 for parents; $250 for Elders). Data collection and analysis were done concurrently to attain reliability and validity, [53] allowing the monitoring of progress of the interviews and to permit follow-up of ideas emerging from the data [54]. This process was done until no new information was generated from the interviews, suggesting data saturation [55].

### Data analysis

We used an inductive thematic analysis approach [56] to explore patterns and themes across the data. Analysis was conducted by LK and comprehensively reviewed and confirmed by SS and then the entire research team. Interviews were professionally transcribed then audio verified to ensure accuracy and familiarity with the data [56]. Transcripts were uploaded to NVivo, [57] an advanced storage-code-and-retrieval software program, to facilitate the organization and analysis of the data. Inductive analysis was performed in three phases: coding, categorizing and developing themes. We used a combination of inductive coding [58] and thematic analysis [56] to allow the generation of new codes and categories. Codes were operationally defined to ensure consistent application to all data, clustered according to commonality and placed into categories [58], which were then grouped into initial themes. Field notes were used to document observations and decisions. Descriptive statistics were used to summarize demographic data.

### Rigour

Rigor was enhanced through thick description, and comprehensive review and critique of findings and interpretations by the research team [59]. To help achieve reliability in the data, member checking was conducted during the data collection process by seeking confirmation or clarification of any uncertain comments during the interview [60]. Data were checked between interview participants to determine recurrent patterns of behaviours (e.g., “Other people have told me [this]. Have you had the same experience/Is this your preference?”) [59]. Additionally, several hundred people stopped by our booth during the MMF Annual General Assembly (March 25–27, 2022; approximately 3,000 in-person and online attendees) where we shared preliminary findings via a handout and discussions to check findings. Parents stated they agreed with these findings and no additional adaptations were suggested during this event. Detailed field notes and reflective journaling were used during data collection and analysis to document contextual details, assist with pattern and theme recognition, build an audit trail and examine potential biases [54].

## Results

Thirty-three parents contacted LK about participating in the study. One parent did not meet the inclusion criteria and 13 could not be reached for follow-up after two additional attempts to contact them. A total of 19 semi-structured interviews [54] were conducted by LK (3 of which were Elders). Most parents lived in rural communities (n = 14), with the remainder living in an urban area. Demographic characteristics are in Table 1.

**Table 1:** Parent Demographics (n = 19)

Characteristic	n (%)
Age (Years)	
18–30 years old 31–45 years old 46–55 years old Over 55 years old Prefer not to answer Missing Data	5 (26) 8 (42) 2 (11) 3 (16) 0 (0) 1 (5)
Gender	
Woman / female Man / male Gender that does not align with your sex assigned at birth Transgender Two-Spirit Gender-fluid Non-binary Woman/ female and Genderfluid Not sure/ Questioning Prefer not to answer Missing Data	15 (79) 3 (16) 0 (0) 0 (0) 0 (0) 0 (0) 0 (0) 1 (5) 0 (0) 0 (0) 0 (0)
Highest level of formal education completed:	
Less than Grade 5 Grade 5–10 Grade 11–12 Some University or College University of College Degree Post Graduate Degree such as Masters or PhD Prefer not to answer Missing Data	0 (0) 0 (0) 3 (16) 4 (21) 8 (42) 0 (0) 0 (0) 4 (21)
Relationship support: Do you have a supportive adult in your everyday life (e.g., spouse, common-law partner, other partner/relationship)?	
Yes No Prefer not to answer Missing Data	11 (58) 4 (21) 1 (5) 3 (16)
Yearly Household Income: (i.e. total sum of income from all adults living in house hold)	
Less than 10,000 10,001–30,000 30,001–50,000 50,001–70,000 70,001–100,000 Greater than 100,000 Prefer not to answer Missing Data	0 (0) 1 (5) 5 (26) 2 (11) 1 (5) 4 (21) 2 (11) 4 (21)
Occupational Status	
Unemployed Household worker Casually Employed (Less than 10 h per week) Employed: Part-time (Less than 35 h, more than 10) Employed: Full-time (35 h per week or more) Retired Prefer not to answer Missing Data	0 (0) 0 (0) 0 (0) 3 (16) 11 (58) 0 (0) 1 (5) 4 (21)
Number of Children in the Family	
0 1 2 3 4 5 Prefer not to answer Missing Data	0 (0) 1 (5) 4 (21) 5 (26) 3 (16) 3 (16) 0 (0) 3 (16)
Primary Language Spoken in the Household	
English English and French English and Saulteaux Missing Data	14 (74) 1 (5) 1 (5) 3 (16)
Have you used Emergency Department Services for your Child?	
Yes No Missing Data	11 (58) 3 (16) 5 (26)

Interviews ranged from 40 to 80 min (M = 52.47). Analysis generated four themes: (1) We’re here too; (2) We are not all the same; (3) Finding trustworthy information; and (4) Information needs to be widely available.

### Theme 1: we’re here too

Parents shared their personal experiences and reflections on being Red River Métis and their relationship with Métis culture. They spoke of their pride and strength in being Red River Métis and the cultural values passed down through family or other ways. However, the generational impact of colonial oppression and racism are reflected in some parents’ experiences of family disconnection from Red River Métis identity and culture, and fear of healthcare providers. Additionally, the divisions between Indigenous groups that were created by colonization can be heard in parents’ reports of Red River Métis getting lost among the description of Indigenous Peoples and not having access to the same federally funded, non-insured health programs as First Nations and Inuit. (Table 2)

**Table 2:** Métis pride and experiences

Theme	Parent/ Elder	Quotes	Category
**We’re here too**	Father, 001	“Métis people are proud people, we’re proud to be – especially Manitoba Métis people. I’m pretty proud to be Métis”	Being Métis
	Mother,020	“you get this feeling inside like, right on, you’re representing the Métis culture, kind of a thing.”	Being Métis
	Mother,002	“we’re kind of in the process of redefining or further defining what it means to be Métis and the differences than the other two Indigenous groups of Canada”	Being Métis
	Mother,003	“Métis people in general always kind of fall into the same thing under, just full aboriginal, whereas they kind of get lost in things. They always feel separated from stuff, and nothing is solely for them”	Being Métis
	Mother,006	That personal connection is key. It’s important. We come from a background where culturally we work together. We’re more communal. At least that’s how it was in my upbringing.”	Cultural values
	Mother,020	“…with the Métis everything is about food… Anytime I go to an event we always have the snacks, the lunch, the supper, whatever it may be…when I go visiting around, it’s always about the food at people’s houses…I think us Métis people love our food.”	Cultural values
**We are not all the same**	Mother,004	“it’s hard to find that trustworthy source, right, and most people, being Métis, you want to see some Indigenous in a pamphlet before you give it any attention.”	Reflecting Métis culture
	Mother,020	“Anything to do with our Métis culture, I think would catch my attention. Or, like, the beading…the drawing of the flowers and stuff like that. If I was flipping through a page and all of a sudden, I came across that, I definitely would stop…you don’t see it very often. And I think if we seen it more often then I probably wouldn’t think like that, as often.”	Reflecting Métis culture
	Mother,016	“Just from like online books that were written by Métis people. I was looking at a cookbook and they have a lot of Métis beadwork and artwork and sashes…It’s like the artwork would really help in terms of like getting someone interested in it, especially from the Métis community.”	Reflecting Métis culture
	Mother,018	“… I don’t represent myself as one person…It doesn’t matter the colour of your skin, it’s who we are, but we have to go beyond the thought of that. We have to include, because if we include other nationalities and then it will look better upon everyone else, because maybe this family of a different culture/nationality will want to use it, because hey, it includes us. We don’t feel left out or, like that’s how I view it anyways.” “if I came upon a thing like that and I only seen say Caucasians, I would feel so, like, that’s not for me because I don’t see anyone of my skin colour or of my, like, my kids don’t have blue eyes, blond hair and things like that.”	Reflecting Métis culture
	Mother,011	“not everyone has a vehicle to go to the hospital, so, maybe creating that, like, I went with my mom on our health authority bus. Like, that would be something that’s more of a norm.”	Reflecting Métis culture
	Mother,003	“…if you’re doing videos for Métis families, Métis names and something that they can relate to and that they’re like, oh look, that’s my daughter’s name or that’s my family members name in there.”	Reflecting Métis culture
	Elder,012	“it is going to be lost because we’re getting old…I’m trying to teach my [relatives] but it’s like they’re not interested…There are some that will come to see me; they want to learn…I just take them to the patch, right, where I get the medicine from…I’m not hiding it or anything if somebody wants to…To pass it on because it could save a person’s life or make their life better.”	Acknowledging traditional medicines
	Mother,006	“many of our Indigenous languages are oral …an auditory resource that would be amazing…you might know all the words and being able to carry on a full conversation in your traditional language, but not know how to read a pamphlet about medical problems.”	Language
	Mother,012	“they [Métis] all speak English. We might as well say we lost our language already.”	Language

#### Being Red River Métis

Four parents spoke of their pride in being Red River Métis, seeing Red River Métis succeed and seeing Red River Métis culture in books or on TV. Three parents also spoke about efforts to define and celebrate Red River Métis culture, such as programs offered by the MMF (e.g., beading classes, parent groups). However, parents highlighted how scarce Red River Métis-specific resources are: ***“****There’s a lot of great resources that they put out targeting specifically First Nations stuff or Inuit. But we’re kind of here too. We’re more urban usually. We kind of blend in a little bit. But visible too… we fall in that gap because yes, we’re seen as Indigenous people, but we don’t have access to the same programming”. [Mother, 006]*

One parent talked about being disconnected from her Métis identity as a child: *“we just grew up in that generation where it’s just like, you pass for a white person…So it’s all new to me too.” [Mother, 010]* An Elder commented on their childhood experiences of racism in rural Manitoba: *“The Métis family were treated like second class citizens… there was no one that I would have trusted to go and talk to, none at all. But I don’t think that’s the case these days.”[Elder, 017]* Another parent shared the generational impact of racism on her own healthcare experiences, including feeling scared when health professionals would visit her home: *“Generationally it’s engrained. Whether we know it or not… And right now, my people have no reason to respect the medical field or the government. It’s got to be earned now.*“*[Mother, 006]*.

#### Cultural values

During discussions on how resources could be culturally adapted, many parents reflected on their Red River Métis cultural values, including personal connection, humour/laughing together, working together, and gathering over food. As one parent described: *“That personal connection is key. It’s important. We come from a background where culturally we work together.”[Mother, 006]* Similarly, another parent shared the importance of food in bringing people together: “…*with the Métis everything is about food… Anytime I go to an event we always have the snacks, the lunch, the supper… even when I go visiting around, it’s always about the food.” [Mother, 020]*.

### Theme 2: we are not all the same

Red River Métis parents have unique and diverse experiences within different regions of Manitoba. Parents shared ways the existing resources could be changed to reflect Red River Métis families and culture better. They described changes to the artwork or graphics that might help to capture their attention, without appearing tokenistic, and making information about traditional medicines, healers and Elders more accessible. Additionally, parents discussed the need for resources to be available in languages other than English, the loss of Indigenous languages, and their interest in reconnecting with these languages.

#### Reflecting Red River Métis culture

Five parents did not think any changes were needed to the existing resources’ artwork or graphics. Some felt the artwork was fine as it was, the skin and hair colours represented were diverse, and that parents would not need to see Métis artwork to look at a child health resource: *“I love our craftwork but I’m not the person that needs to see like a beading pattern on my pamphlet from the doctor…I want you to tell me the facts and I want you to tell me them in as clear a language as possible.” [Mother, 006]* Two parents warned that attempts to represent cultural groups can appear tokenistic and end up othering a group or individual and seeing them as different from the ‘norm’: *“If we’re talking specifically Métis. There’s a rainbow of us, right. Not all the same. And I think we try to do better with representation but sometimes it feels really just a little bit too tokenistic…why do they always have to be about like the seven teachings or Bannock or hunting? Like one of my favourite ones is just a book about a family setting up for Christmas. They just happen to be Brown. Like, why can’t that exist?” [Mother, 006]*.

Six parents spoke about how seeing Red River Métis culture within a child health resource could help families connect with it. For example, seeing themselves or their culture included in a resource may feel more trustworthy or welcoming, and it may catch their interest because it is not something they see very often. Five parents suggested including artwork that would represent Red River Métis culture, such as flower drawings, beadwork, traditional clothing and the Métis sash. Other suggestions included using Métis names, showing families from different cultural groups to acknowledge that people can identify with multiple groups, and avoiding gender and family stereotypes: *“… I think you could use more variety in like the ages of the caregivers taking care of the children. Especially for like the Métis and all Indigenous communities, we have lots of blended families and aunties and uncles taking care of the kids too”. [Mother, 002]* Another parent wanted to see a variety of homes and foods: *“… my kids, they like a lot of wildlife, like, moose and bears and deer…Like that’s kind of what we eat and kind of what we’re raised on”. [Mother, 011]* This parent also highlighted the importance of including different transport options. For example, limited taxi services, and other transport barriers, particularly within rural areas, may result in families staying in a hotel once discharged from the hospital. Showing ways other than driving a car to the hospital could better represent diverse family situations.

#### Acknowledging traditional medicines

Parents spoke of using traditional medicines or knowing Métis families who do and wanted child health information that acknowledged this use. As one mother described: *“…sometimes the health information can end up being very Westernized and not always the way that Métis people would necessarily start dealing with an illness.” [Mother, 002]* Another parent suggested: *“something like that, if you want some traditional medicine, contact your local healer, this is their name….but like to even include some holistic stuff in there, like, from an Elder, that would be awesome.” [Mother, 007*] However, parents spoke of doctors’ reluctance to discuss traditional medicine. As one parent said: *“It’s a touchy subject. Doctors don’t even touch it.” [Mother, 004]* Similarly, parents did not feel comfortable bringing the subject up with doctors. An Elder shared her mother’s reluctance to tell doctors about their use of herbal medicines: *“my mom used to say, Is that legal to do that, to give herbal medicines? She used to say don’t tell the doctor I gave you that. And yet it’s natural medicine…And you could see the benefits right away…we survived all these years…The only time I think somebody went to the [hospital], is if they had like problems breathing”. [Elder, 012]*

#### Language

Parents were asked if the existing resources should be available in languages other than English. The most frequently cited language was French (n = 13); reasons included to represent Canada’s two national languages and because many Red River Métis speak French. The next most cited languages were Michif (n = 8) and Cree (n = 8), followed by Ojibway (n = 6), Saulteaux (n = 5), and Swampy Cree (n = 1). Thirteen of the parents reported that English was the primary language spoken in their home, with one parent reporting English and Saulteaux were spoken (Table 1). None of the parents indicated they themselves were fluent in an Indigenous language. One parent understood but could not speak Saulteaux.

An Elder shared their experience of forced assimilation within the education system: *“We couldn’t use our language. We had to speak English.” [Elder, 012]* Another Elder explained that “.*most people are the same with me. They’re older people, it [Indigenous language] kind of stopped with them. The youngest… It’s all a foreign language to them now.” [Elder, 019]* Four parents spoke about the loss of Indigenous languages and questioned whether a resource in another language would be useful to families whose main language was English. Some parents spoke of interest in reconnecting with Indigenous languages: *“…if you were trying to encourage Métis community, to do it in Michif… I mean medical terms are complicated, but that might be a fun way to encourage people to read it. There’s lots of people who are trying to reconnect to language that way.” [Mother, 002]* Additionally, one parent suggested having an auditory resource, to reach people who may speak a language but not be able to read it.

### Theme 3: finding trustworthy information

This theme focuses on the relationships and places that are trusted for accessing child health information. Learning about the information sources that Red River Métis families trust can help inform the processes of developing and disseminating this information. Parents shared the people, organizations and places they trust to get child health resources but also emphasized the importance of accessible, safe spaces to find this information. (Table 3)

**Table 3:** Preferences for child health information

Theme	Parent/Elder	Quotes	Category
**Finding trustworthy information**	Mother,007	“I just don’t have the time or the patience to search through the internet and read up on stuff… they [health professionals] say Google it. Well, no, I’m coming to you because I want your opinion on it.”	Communicating with healthcare professionals
	Mother,003	“Something written by professionals, like, a pediatrician or not just a pediatrician but like specialists…. where you can connect to them because they’re from Canada… they practice the same medicine where you live because around the world obviously, they practice it differently and they have different techniques for different things.”	Communicating with healthcare professionals
		“it gets confusing where one person will say one thing and another. It seems in general it’s just very difficult to find what answer is right, what one is wrong”	
	Elder, 017	“A lot of people are not computer savvy or they don’t know anybody that might be able to help them. So, they’re in the dark, who do they go to? So, if they’re really sick, they go to emerg. Or urgent care.“	Communicating with healthcare professionals
	Mother,006	“I would want it to be curated and monitored by people who have medical training or at least knowledge… I don’t want Wikipedia where everybody can go and edit it…Or at least an actual registered organization that it is, is not spewing rhetoric or false studies.”	Communicating with healthcare professionals
		“…I think if you have a person that you can communicate with that you can form a trusting connection with, even if they’re just helping you navigate and talk to this person and talk to that person…I think culturally speaking is, is very important, especially in the climate we’re in right now. Right. We want reconciliation. We have to acknowledge that there’s a problem in our healthcare system. And it’s a lack of understanding and respect that creates and promotes fear along with the past”	
	Mother,004	“hearing doctors who, like all these high words of vocabulary ask you of things you have no idea what they’re saying or anything. So, it’s no comfort. Knowing they’re trying to explain to you but you don’t even understand what they’re saying. So, a lot of times I would reach out to social media for this feedback.”	Communicating with healthcare professionals
	Mother,007	“our professionals are busy and they’ve got to see their patients and get through the day, but, the one’s that take the time to listen, that makes you feel really important and that what’s going on with you matters.”	Communicating with healthcare professionals
**Information needs to be widely available**	Mother,006	“…most people have smartphones, but there is something said about holding something in your hand. About physically being handed something that has words on it or a number or a website that oh, this can help…It’s comforting to touch it.”	Digital formats
	Mother, 009	“I like how when you go onto social media, it’s there right when you need it.”	Digital formats
	Mother,018	“Facebook for parents, because I like to have that interaction with an actual parent…then you don’t feel so alone…There’s a lot of people like me that have kids that deal with things like that…it’s the connection.”	Digital formats
	Mother,009	“I really like videos. I’m just more of a visual learner that way.”	Digital formats
	Mother, 004	“when my son was heavy breathing, I didn’t understand what was going on so going on social media and just looking it over or even just making a post on Facebook and asking has anyone experienced this and then you get feedback immediately”.	Digital formats
	Mother,003	“… a video would be fast, so they could be like, okay, the kid is sick, this is what we should do instead of panicking and running to an urgent care or an emergency.”	Digital formats
	Mother, 011	“… videos are helpful and kind of easy snippets to watch. I like reading stuff and holding it actually and referring back to it too though.”	Paper formats

#### Communicating with healthcare professionals

Of the 13 parents that reported turning to family and friends for health information when their child is sick, 11 stated that their family or friends worked in health care. Parents wanted to get health information from healthcare professionals, in particular when they did not know where to look for this information or were not comfortable looking up information online. However, the *way* healthcare professionals share this information is important. While most parents wanted to access trusted information from healthcare professionals, they also highlighted the difficulties they can face when engaging those healthcare professional sources. Two parents reported witnessing discrimination from healthcare professionals, which can deter people from trusting and reaching out to them: *“racial discrimination deters people from calling [for medical help]…if I’m going to something and I feel unwanted…., I’m definitely not gonna be going back.” [Mother, 010]* Parents also spoke about difficulties getting clear answers to their questions. For example, they described turning to social media when doctors use medical jargon, and feeling “*small because they don’t understand what they’re talking about.” [Mother, 018]* Five parents wanted access to one trusted place, whether in-person or online, to find information and also help when they feel overwhelmed about how best to navigate their child’s health care.

#### Sharing information

Parents commented they would trust information from an Indigenous source, and if it was from organizations (i.e., not an online forum or a personal opinion), such as a government body or a university that shares research and cites sources. Suggested places to share child health information included schools, daycares, community centres, baby clinics, babysitting courses, social workers, doctor’s office, provincial health department, public health offices, early childhood programs and the MMF. For example, it was suggested child health information could be included within an organization’s website, emails/newsletters, and existing resource kits (e.g., MMF’s Little Métis Box [https://www.mmf.mb.ca/early-learning-child-care], an activity kit offered to families with children aged zero to six to explore Red River Métis culture and history together). Parenting groups/classes were also suggested: *“ … come in and talk about stuff and talk about the website and talk about things that are in the website, … give them pamphlets… just include the parents in stuff like that so they know that it’s out there, because maybe some people don’t have access to a computer or access to a phone and access to anything.” [Mother, 018]*.

### Theme 4: information needs to be widely available

This theme describes parents’ accounts of the information formats (i.e., the way information is presented) they use or would prefer to access. While most parents reported looking for child health information online, they also stressed the continued need to provide multiple options, including information sheets. Parents seek information in different contexts. Widely available information may help worried families decide whether to travel long distances and/or incur the costs of seeking medical care for their child. For example, one parent reported: *“information needs to be put out widely, not just, like, oh, go to the hospital…For me to take my children to the hospital it’s an hour drive. To go to [city/town], is just about two hours from where I live. And then if we have to call in an ambulance, that costs us like $500 something. Sometimes it is what it is, but that’s why I say, information anywhere would be great.” [Mother, 018]*.

#### Digital formats

Searching online for child health information was reported by 17 parents. Google was the most cited online source (n = 12) but its limitations were acknowledged: *“… you Google for your splinter, and you’re dying of cancer next week in your head. And when it’s your child, that’s dangerous thinking.” [Mother, 006]* Social media was the next most cited online source (n = 8). Parents described preferring the succinct and instant format of social media and being able to connect with community groups and other families. Two parents wanted easy access to an online assessment that could help them make decisions on when and where to seek medical care for their sick child: “*just in one location…Because it’s that step before having to make that decision whether to call your family doctor or to go to the urgent care versus the emergency”. [Mother, 003]* Online information sessions were suggested by two parents. For example, a class to *“go through some of these things that are on the website and how to react.” [Mother, 005]*. Three parents mentioned getting information via email would be helpful and six parents liked videos. While videos were described as being quick to watch, web pages were suggested when parents have more time: *“… scrolling through is really nice. If you’re sitting in a waiting room and you’ve got the time then it’s something that you can read to expect what’s going to happen next for treatment versus what you’re going to do at home for treatment.” [Mother, 003]*.

#### Paper formats

Parents stressed the importance of having multiple ways to access child health information. For example, some families cannot access the internet, cell phone or landline to find information. While most parents went online, many valued having a hard copy format that would direct them or remind them of this online information: *“an information sheet with, oh, if you want to look on YouTube, you can watch this video. If you want to look on the website, go to Google, search for this….I’d rather grab a pamphlet and read it like that or talk to somebody who has experienced it…” [Mother, 007]*.

## Discussion

Parents provided tangible ways to modify existing KT tools for Red River Métis families, including adding information on how to access Elders, healers and/or traditional medicines and showing different family structures, transport, living situations, Métis names and social gatherings with food. While modifying the appearance of a resource can be part of the cultural adaptation process, [61] there must also be an explicit focus on decolonizing and anti-racist approaches [62] during the KT process. Co-creating resources *with* Red River Métis families and privileging their lived experiences and perspectives to guide the adaptation process is in itself a decolonizing approach [44]. This approach creates a space to decolonize the health information we provide (i.e. value Métis’ cultural contexts, experiences and knowledge and support the creation of strengths-based rather than deficit-based resources) and in turn promote anti-racist, holistic child health care [44].

We focus our discussion on key considerations for developing and/or adapting child health information for Red River Métis families: (1) supporting the revitalization of Métis language and culture: (2) building relationships; and (3) context.

### Child health resources can support revitalization of Métis language and culture

Red River Métis pride was prominent in the results. While the results highlight mixed messages around the relative value and benefit in providing Red River Métis specific resources, it cannot be understated that this is a function of colonization [63]. Métis lived under a reign of terror in being Métis [64] and subsequently learned to hide their identity, language, deemphasize their First Nations or Métis heritage and emphasize Euro-Canadian heritage to reduce exposure to racism, social injustices and health inequalities [35]. Métis history has been denied or silenced for centuries and colonization continues to impact the relationship Métis have with their identity and culture, including connection to language, key people (e.g., Elders), cultural teachings and traditional medicines [35, 63]. Generations of people who may not have formally identified as Métis have come forward with increased pride and desire to identify as Métis, in part due to the formal development of Métis government structures and increased recognition of Métis in Canadian court cases [35, 44, 63].

Cultural health, which is defined as expressing and celebrating one’s identity and place in the world, is important for health and well-being [65]. There is a gap in research focusing on the distinct experiences of Métis families [66]. Resources can help to counter a legacy of colonization by: (1) focusing on the strengths of Métis (i.e., traditions, ways people support each other in community, community-driven solutions); (2) promoting the understanding that Métis people, organizations, and communities have the knowledge and expertise to identify and address their own concerns; and (3) supporting the cultural identity of Métis through knowledge sharing and documenting these important teachings for future generations [67].

There is a resurgence towards language revitalization, and information resources in Michif or another of the Métis languages could support such an initiative. The Michif language was once common in Métis communities and is now considered endangered, with fewer than 1000 people speaking it. Language revitalization efforts promote individual and community healing [63, 65, 68] and there was interest among the parents to learn an Indigenous language. The MMF is promoting Michif through access to language resources and lessons at the Louis Riel Institute [69, 70]. Inserting Michif, and/or another language (e.g., Saulteaux or Cree) alongside English text in a child health resource could be a favorable way to integrate language that would help both adults and children learn together in a more fun and engaging way. Working with language experts will be important to ensure content is interpreted and translated correctly, for example, dialect and associated nuances in the messaging are not lost [67].

Parents wanted information on how to connect with Elders, Indigenous healers and traditional medicines, and this has also been reported in other studies with Indigenous families looking for child health information [71–79]. Resources can acknowledge and respect the use of these services by including clear signposting to more information, advice and support available. Healthcare providers who may not be able to incorporate traditional medicines and healing practices into treatment plans could share the resources to demonstrate their respect and support of Métis cultural resources and their roles in health and healing [66, 75, 77]. However, challenges in accessing these supports, such as not being available in local communities, [80] must also be considered in any resource development.

### Relationships are key for sharing trusted information

Information is just one part of a health communication process that can also involve interactions with healthcare professionals, family and friends [81]. Relationships are key for information sharing and tied to trust. Kinship and social networks are sources of information and comfort for parents in this study and these can be effective approaches for knowledge translation [82]. However, Red River Métis families also want child health information and advice from healthcare professionals, which is in line with extant literature [66, 83, 84]. The tension that some parents experienced racism in the health system and therefore distrust health professionals is not incongruent with trusting health professionals to provide accurate health information. Parents who are worried about their sick child will do whatever they can to help them, including turning to a health provider for advice. The relationship with these health providers matters, and studies have reported Indigenous families are less likely to trust healthcare providers’ advice [77, 78] and will seek care elsewhere [77] if they feel their perspectives and unique knowledge about their child is being dismissed. Culturally appropriate health care, of which clear information is a vital component, offered in an environment free of racism and stereotypes, can improve health outcomes and quality of care; unfortunately, this type of care is not available to everyone [85]. Many healthcare providers lack training in culturally appropriate approaches [86]. We recently conducted a scoping review that highlighted actions individual healthcare providers can take to improve Indigenous families’ access to meaningful child health information: listening to patients/families, including them in decision-making, giving honest information, and sharing information within families’ communities [66]. Additionally, Métis health professionals are trusted for health information and an important component of cultural safety [65, 87].

Red River Métis parents in this study emphasized the importance of accessible, safe spaces to find child health information, including local schools, community centres, healthcare organizations and the MMF. A 2020 guide to producing health information highlighted schools and teachers as reliable sources of health information for families [81]. Additionally, accessing information closer to home has been a preference for Indigenous families in other studies [71]. People in Manitoba have access to a free, phone-based program for access to general health advice from registered nurses (https://www.gov.mb.ca/betterhealth/index.html). However, accessing this service can be challenging in areas where there is little to no cell coverage and families may have to drive some distances to get a signal. Investing time to build relationships with healthcare, educational and community organizations is critical to the dissemination of accessible and trusted child health resources [88–91].

### Context matters

Findings demonstrate a variety of sources and formats (e.g., via healthcare providers vs. social connections; online vs. paper-based) that can make it challenging to know where to begin with the resource development process. Different people have different experiences and opportunities, which shape their needs, concerns, challenges, interests and preferences for accessing health information. Effective health communication is not generic and the variety in preferences is reflective of the different contexts in which Red River Métis live. We can try to serve these varied needs with layered approaches to communication and sharing of child health information. For example, printed information can signpost to more online details. Information on web pages can be supported by replicating the information on a printable PDF. Healthcare settings, such as waiting rooms, could have computers with internet for families to access health information before or after their visit. These low-cost, simple-to-implement ideas can be practical ways to share knowledge and also have also been suggested in other studies [92, 93]. Additionally, incorporating an intersectionality lens within the KT process can promote the understanding that people are shaped by different social locations or categories (e.g., gender, race, Indigeneity, class) [50, 94, 95]. Intersectionality-informed research can help to advance the understanding of the commonalities and differences among Red River Métis parents seeking health information, as well as highlight the similar and different experiences of parents across social categories.

Contextualization is key to successfully utilize approaches according to the specific health information or decision-making needs; for example, making a decision to seek care; receiving information after a diagnosis or about a treatment; looking for emotional support versus specific information. Recognition of contexts is also an essential part of KT [96]. However, KT efforts within health research are deeply embedded in Western science approaches [97]. A scoping review of promising and wise KT practices in the Indigenous health field reported meaningful involvement of families and other users of the health information is an effective KT method [82]. It is also fundamental to decolonization [98]. Co-creation of child health resources *with* Red River Métis families and communities can ensure the KT process is planned and implemented in context, and that the shared knowledges will reflect the local protocols, conditions, history, culture, languages and worldviews of the community [82].

### Strengths and limitations

To the best of our knowledge, this is among the first studies that aims to understand Red River Métis parents’ experiences and preferences for seeking child health information when their child is acutely ill. It was conducted in partnership with the MMF to help address the lack of child health information for Red River Métis families. Additionally, we shared and verified study findings with Red River Métis citizens attending the MMF annual general assembly. However, the study does have some limitations. It was conducted during the COVID-19 pandemic, which restricted the ability to conduct in-person interviews or focus groups, which may have impacted the engagement of parents who preferred this option. Lastly, parents were not asked to demonstrate Red River Métis status (e.g., showing a citizenship card) and it is therefore possible that study participants may not fall under the official definition of Red River Métis citizenship. However, as recruitment was done through the MMF, it is assumed that all participants had some level of involvement in the Red River Métis Nation.

## Conclusion

There is a lack of child health information aimed at Red River Métis families, and the development of this information can support their information needs and preferences and the revitalization of Red River Métis culture and language. Parents suggested ways to modify the existing KT tools, including incorporating Michif or another of the Métis languages, adding information on how to access Elders, healers and/or traditional medicines, as well as showing different family structures, transport, living situations, Métis names and social gatherings with food. Kinship and social networks are sources of information and comfort for parents in this study however they also want child health information and advice from healthcare professionals. Investment of time to build relationships with the MMF, Red River Métis organizations and communities is needed to co-create child health resources with Red River Métis families and disseminate these resources effectively. Gaining a better understanding of the information-seeking experiences and preferences of Red River Métis families can expose and challenge existing colonial processes perpetuating inequitable access to child health information. This research is a critical step in addressing an identified need by the MMF for Red River Métis families to have culturally relevant and meaningful resources available when their children are sick, and in the pursuit of equitable care for all children in Canada.

## Data Availability

Data are available upon reasonable request from the corresponding author.

## List of abbreviations

MMF: Manitoba Métis Federation
KT: knowledge translation
